# Chinese yam polysaccharide promotes jejunal development, antioxidant defense, and mucosal immunity in weaned rats

**DOI:** 10.3389/fvets.2026.1721855

**Published:** 2026-02-03

**Authors:** Chuanyan Che, Ya Yang, Hong Hu, Mengmeng Jin, Shenghe Li, Ahmed H. Ghonaim, Mai G. Hopo, Changsheng Jiang, Man Ren

**Affiliations:** 1 Anhui Provincial Key Laboratory of Animal Nutritional Regulation and Health, College of Animal Science, Anhui Science and Technology University, Chuzhou, China; 2 Anhui Engineering Technology Research Center of Pork Quality Control and Enhance, Anhui Science and Technology University, Chuzhou, China; 3 National Key Laboratory of Agricultural Microbiology, College of Animal Sciences and Veterinary Medicine, Huazhong Agricultural University, Wuhan, China; 4 Desert Research Center, Cairo, Egypt; 5 Department of Aquatic Animal Medicine, College of Fisheries, Huazhong Agricultural University, Wuhan, Hubei, China

**Keywords:** antioxidant defense, Chinese yam polysaccharide, intestinal barrier, intestinal morphology, mucosal immunity

## Abstract

**Introduction:**

Chinese yam polysaccharide (CYP), a heteropolysaccharide composed of mannose, xylose, arabinose, glucose, and galactose, has been reported to exhibit immunomodulatory and antioxidant properties. However, its role in regulating intestinal development and mucosal barrier function remains incompletely understood. This study investigated the effects of dietary CYP supplementation on antioxidant status, jejunal morphology, digestive function, and mucosal immunity in weaned rats.

**Methods:**

Thirty specific-pathogen-free male Sprague–Dawley rats (initial body weight: 50.16 ± 0.50 g) were randomly assigned to three dietary groups (n = 10 per group) for a 28-day feeding trial: a control group fed a basal diet and two treatment groups fed the basal diet supplemented with 0.1% or 0.5% CYP. At the end of the experiment, blood and jejunal tissues were collected for biochemical, histomorphological, and molecular analyses.

**Results:**

Compared with the control group, supplementation with 0.5% CYP significantly reduced plasma malondialdehyde (MDA) concentrations (*P* < 0.05) and increased total antioxidant capacity (*P* < 0.01), which was associated with activation of the MAPK ERK1/2–Nrf2 signaling pathway. Both 0.1% and 0.5% CYP supplementation significantly increased jejunal villus height and the villus height-to-crypt depth ratio (*P* < 0.05), while reducing intraepithelial lymphocyte numbers (*P* < 0.05). Moreover, rats receiving 0.5% CYP exhibited significantly higher activities of lactase, sucrase, and maltase, along with increased jejunal immunoglobulin A (IgA) and β-defensin levels (*P* < 0.05 or *P* < 0.01). Additionally, CYP supplementation at both inclusion levels markedly upregulated the expression of Claudin-1 and Mucin-1 (*P* < 0.01).

**Discussion:**

Collectively, these findings demonstrate that dietary CYP enhances systemic antioxidant defense, promotes jejunal structural development, and strengthens intestinal mucosal barrier function in weaned rats.

## 1 Introduction

The intestinal tract is a critical organ responsible for both nutrient digestion and immune defense (1). While facilitating nutrient absorption, it is continuously exposed to a wide range of dietary antigens and microbial challenges (2). Consequently, the integrity of the intestinal mucosal barrier is essential for maintaining intestinal homeostasis. Disruption of this barrier can result in enteritis, bacteremia, diarrhea, and in severe cases, mortality (3). Young animals possess immature intestinal structures and immune systems, rendering them particularly susceptible to intestinal inflammation and post-weaning diarrhea (e.g., in piglets), which leads to considerable economic losses in animal production systems. With increasing restrictions on the use of antibiotic growth promoters, there has been growing interest in functional feed additives. Among these, plant-derived polysaccharides, such as those extracted from *Astragalus, Echinacea*, and *Lentinus*, have attracted attention due to their immunomodulatory and gut-protective properties (4, 5).

Chinese yam (*Dioscorea opposita* Thunb.) has been consumed for thousands of years in China as both a food and a traditional medicinal herb. In traditional Chinese medicine, it is believed to “invigorate the spleen, tonify the lung, strengthen the kidney, and nourish essence” (6, 7). Chinese yam polysaccharide (CYP), one of its principal bioactive components, is composed mainly of mannose, xylose, arabinose, glucose, and galactose (8). Increasing evidence indicates that CYP exerts a wide range of biological activities. For instance, a Chinese yam polysaccharide–copper complex improved growth performance, carcass traits, antioxidant status, serum biochemical parameters, and immune function in broilers (9). In addition, CYP and sulfated Chinese yam polysaccharides (SCYP) have demonstrated anti-inflammatory effects and the ability to modulate gut microbiota composition (10). CYP has also been shown to attenuate lipopolysaccharide-induced inflammation in a Caco-2/RAW264.7 co-culture model (11), regulate muscle development-related gene expression in broilers (12), and alleviate cancer-related fatigue in mice, potentially through modulation of oxidative stress, inflammatory responses, and energy metabolism (13). Despite these findings, the effects of CYP on the intestinal barrier function and the underlying molecular mechanisms remain insufficiently characterized.

Intestinal epithelial cells exhibit high metabolic activity, which inevitably leads to the generation of reactive oxygen species (ROS). When ROS are not efficiently eliminated, they can damage mitochondria, cellular membranes, and other organelles, thereby impairing epithelial renewal and triggering mucosal injury and inflammation (14, 15). Based on the reported antioxidant and immunomodulatory properties of CYP, we hypothesized that CYP may protect intestinal barrier integrity by enhancing intestinal antioxidant capacity. The mitogen-activated protein kinase/extracellular signal-regulated kinase–nuclear factor erythroid 2–related factor 2 (MAPK/ERK–Nrf2) signaling axis plays a pivotal role in cellular defense against oxidative stress and inflammation, contributing to the maintenance of intestinal homeostasis (16). Previous studies have demonstrated that phosphorylation of ERK1/2 can activate Nrf2-mediated antioxidant responses (17, 18). Moreover, various natural bioactive compounds, including polyphenols, flavonoids, and polysaccharides, have been reported to enhance intestinal antioxidant and anti-inflammatory defenses by modulating MAPK/ERK and Nrf2 signaling pathways (19, 20).

Whether CYP, as a natural plant polysaccharide, exerts protective effects on the intestinal mucosal barrier through modulation of the MAPK/ERK–Nrf2 signaling pathway remains unclear. Therefore, the present study used a weaned rat model to investigate the effects of dietary CYP supplementation on jejunal morphology, mucosal barrier function, digestive enzyme activity, and the expression of oxidative stress–related signaling proteins. To our knowledge, this is the first study to systematically demonstrate that Chinese yam polysaccharide enhances jejunal development during the weaning period through coordinated regulation of antioxidant defense and mucosal immunity.

## 2 Materials and methods

### 2.1 Experimental design and sample collection

All animal experimental procedures were reviewed and approved by the Animal Ethics Committee of Anhui Science and Technology University (protocol number 2023088) and were conducted in accordance with the “Guidelines for the Care and Use of Test Animals” of Anhui Province.

Thirty specific-pathogen-free (SPF) male Sprague–Dawley (SD) weaned rats, with an initial body weight of 50.16 ± 0.50 g, were obtained from the Zhejiang Academy of Medical Sciences. Rats were randomly assigned to three experimental groups (*n* = 10 per group). The control group received a basal diet, while the two treatment groups were fed the basal diet supplemented with either 0.1 or 0.5% Chinese yam polysaccharide (CYP; purity 70%; Shaanxi Zhongxin Biotechnology Co., Ltd., China). The experimental diets were formulated by thoroughly mixing CYP with the basal diet to ensure uniform distribution. All rats were housed in a controlled environment maintained at a temperature of 20–22 °C, relative humidity of approximately 50%, and a 12 h light/12 h dark cycle. Animals had *ad libitum* access to feed and water throughout the 28-day experimental period.

At the end of the feeding trial, rats were euthanized by diethyl ether anesthesia. Whole blood samples were collected and centrifuged at 3,000 × g for 10 min at 4 °C to obtain serum, which was stored at −80 °C for subsequent biochemical analyses. The mid-jejunum was immediately excised, gently flushed with ice-cold saline, and divided into two portions. One portion was fixed in 4% paraformaldehyde for histomorphological and immunohistochemical analyses, while the other portion was snap-frozen in liquid nitrogen and stored at −80 °C for molecular and protein analyses.

### 2.2 Tissue staining and histomorphological observation

Jejunal samples fixed in 4% paraformaldehyde were dehydrated, embedded in paraffin, and sectioned at a thickness of 5 μm. Sections were deparaffinized in xylene and rehydrated through a graded ethanol series (100%, 95%, 85%, and 70%). Hematoxylin and eosin (H&E) staining was performed using a commercial staining kit (Solarbio, Beijing, China) following the manufacturer's instructions. Stained sections were examined under a light microscope equipped with a pathological image acquisition system. Villus height and crypt depth were measured using Image-Pro Plus 6.0 software (Media Cybernetics, USA). Only intact, well-oriented villi were selected for quantitative analysis, and the villus height-to-crypt depth ratio was calculated accordingly.

Intraepithelial lymphocytes (IELs) were identified as small, round cells located between intestinal epithelial cells near the basement membrane, characterized by deeply stained nuclei and minimal cytoplasm. IELs were counted in randomly selected intact villi under 200 × magnification, and the average number was used to assess lymphocyte density within the epithelial layer.

### 2.3 Determination of plasma biochemical and antioxidant Indices

Plasma biochemical indicators, including blood ammonia (BA) and non-esterified fatty acids (NEFA), as well as antioxidant parameters such as total antioxidant capacity (T-AOC) and malondialdehyde (MDA), were measured using commercial assay kits (Nanjing Jiancheng Bioengineering Institute, Nanjing, China) in strict accordance with the manufacturer's instructions. Detailed information on the assay kits used for biochemical and antioxidant measurements is provided in Table 1.

**Table 1:** Kits for plasma biochemical and antioxidant index determination.

**Kit name**	**Catalog no**.	**Manufacturer**
Blood ammonia assay kit	A086-2-1	Nanjing Jiancheng Bioengineer Institute, PRC
Non-esterified Free fatty acids assay kit	A042-2-1	
Total antioxidant capacity assay kit	A015-2-1	
Malondialdehyde assay kit	A045-2-2	

### 2.4 Determination of digestive enzymes activities and immune protein contents

Jejunal tissue homogenates were prepared for the determination of digestive enzyme activities and immune-related protein levels. The activities of lactase, sucrase, and adenosine triphosphatase (ATPase), as well as the concentrations of nuclear factor erythroid 2–related factor 2 (Nrf2), immunoglobulin A (IgA), defensin beta 1 (DEFβ1), interleukin-1β (IL-1β), and interleukin-6 (IL-6), were quantified using commercial kits (Nanjing Jiancheng Bioengineering Institute, Nanjing, China; Jianglai Biotechnology Co., Ltd., Shanghai, China) according to the manufacturers' protocols. Details of all assay kits are listed in Table 2.

**Table 2:** Kits for determination of digestive enzymes activities and immune protein contents.

**Kit name**	**Catalog no**.	**Manufacturer**
Lactase assay kit	A082-1-1	Nanjing Jiancheng Bioengineer Institute, PRC
Sucrase assay kit	A082-2-1	
Maltase assay kit	A082-3-1	
Na^+^K^+^-ATPase assay kit	A070-2-2	
Ca^2+^Mg^2+^-ATPase assay kit	A016-2-2	
Nrf2 ELISA kit	H319-1-2	
Rat Immunoglobulin A (IgA) ELISA Kit	JL12859-96T	Jianglai Biotechnology Co., LTD
Rat Defensin Beta 1 (DEFβ1) ELISA Kit	JL29995-96T	
Rat Interleukin 6 (IL-6) ELISA Kit	JL20896-96T	
Rat Interleukin 1 Beta (IL-1β) ELISA Kit	JL20884-96T	

### 2.5 Western blot analysis

Total proteins from jejunal tissues were extracted using RIPA lysis buffer supplemented with 1% phenylmethylsulfonyl fluoride (PMSF; Beyotime, Shanghai, China) on ice. Protein concentrations were determined using a bicinchoninic acid protein assay kit (Beyotime, China). Equal amounts of protein (20 μg per sample) were separated by 10% SDS–polyacrylamide gel electrophoresis and transferred onto polyvinylidene difluoride membranes (Millipore, USA). Membranes were blocked with 5% bovine serum albumin in Tris-buffered saline containing 0.1% Tween-20 for 2 h at room temperature and then incubated overnight at 4 °C with primary antibodies (1:2,000 dilution). After washing, membranes were incubated with horseradish peroxidase (HRP)-conjugated secondary antibodies (1:5,000 dilution) for 2 h at room temperature. Protein bands were visualized using an enhanced chemiluminescence detection kit (Solarbio, Beijing, China) and imaged with a ChemiDoc™ imaging system (Bio-Rad, USA). Band intensities were quantified using ImageJ software (NIH, USA) and normalized to β-actin. Antibody details are summarized in Table 3.

**Table 3:** Antibodies used in this study.

**Antibodies**	**Manufacturer**	**Catalog no**.
β-Actin Mouse mAb	Cell Signaling Technology, Inc	3,700
Claudin-1 Rabbit mAb		13,255
MUC1 Rabbit mAb		14,161
MAPK (Erk1/2) Rabbit mAb		4,695
Phospho-MAPK (ERK1/2) Rabbit mAb		4,370
HRP-conjugated Goat anti-Rabbit IgG (H+L)		7,074
HRP-conjugated Goat anti-Mouse IgG (H+L)		91,196

### 2.6 Immunohistochemistry (IHC)

Paraffin-embedded jejunal sections were deparaffinized and rehydrated, followed by antigen retrieval in 10 mM citrate buffer (pH 6.0) using microwave heating. After cooling, sections were washed with phosphate-buffered saline. Endogenous peroxidase activity was blocked with 3% hydrogen peroxide for 12 min at room temperature. Sections were then incubated with 10% normal goat serum for 30 min to block non-specific binding. Sections were incubated overnight at 4 °C with rabbit anti-Claudin-1 or anti-Mucin-1 primary antibodies (1:500 dilution), followed by incubation with biotinylated goat anti-rabbit IgG secondary antibody (1:500 dilution) and HRP-labeled streptavidin (1:500 dilution). Immunoreactivity was visualized using diaminobenzidine, and nuclei were counterstained with hematoxylin. Stained sections were examined under a light microscope, and representative images were captured for analysis.

### 2.7 Statistical analysis

All data are expressed as mean ± standard deviation (SD). Statistical analyses were performed using one-way analysis of variance (ANOVA) in SPSS 20.0 software (IBM Corp., USA). When significant differences were detected, Duncan's multiple range test was applied for *post hoc* comparisons. Values with different lowercase letters indicate significant differences (*P* < 0.05), different uppercase letters indicate highly significant differences (*P* < 0.01), and identical letters indicate no significant difference (*P* > 0.05).

## 3 Results

### 3.1 CYP enhances the antioxidant capacity of rats

As show in Table 4, dietary supplementation with 0.5% CYP significantly reduced serum MDA levels compared with the control group (*P* < 0.05). Both 0.1 and 0.5% CYP markedly increased serum T-AOC levels (*P* < 0.01). Additionally, supplementation with either dose of CYP significantly decreased serum BA concentrations (*P* < 0.01). These findings suggest that CYP supplementation enhances antioxidant capacity and supports hepatic function in rats.

**Table 4:** Effects of CYP on antioxidant capacity and hepatic function in rats.

**Items**	**CON**	**0.1% CYP**	**0.5% CYP**	***P-*value**
MDA (nmol/ml)	2.89 ± 0.41^a^	2.16 ± 0.11^ab^	1.76 ± 0.27^b^	0.03
T-AOC (U/ml)	4.22 ± 0.29^Bb^	6.82 ± 0.35^Aa^	6.88 ± 0.81^Aa^	< 0.01
BA (μmol/ml)	269.07 ± 24.20^Aa^	198.10 ± 11.45^Bb^	127.86 ± 16.08^Cc^	< 0.01
NEFA (μmol/ml)	1,473.50 ± 56.01	1,517.54 ± 109.55	1,784.56 ± 188.30	ns

Within each row, values marked with different lowercase letters differ significantly (*P* < 0.05), those with different uppercase letters differ highly significantly (*P* < 0.01), while identical letters indicate no significant difference (*P* > 0.05).

**Figure 1:**
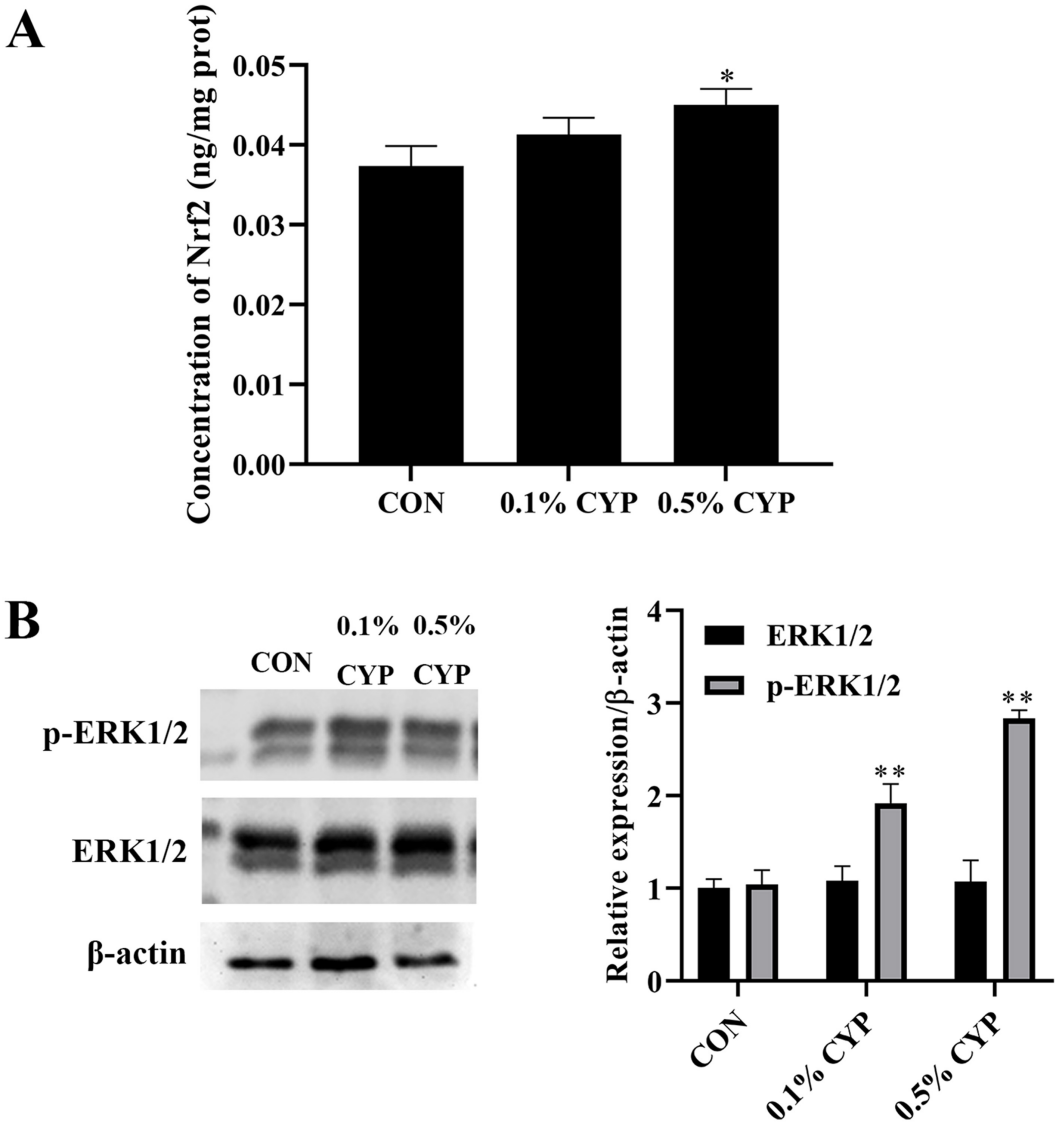
CYP enhances antioxidant capacity in the rat jejunum. **(A)** The concentration of Nrf2 in the jejunum of rats. * denotes *P* < 0.05 vs. control group; **(B)** CYP actives the MAPK ERK1/2 signaling pathway in the jejunum. ** denotes *P* < 0.01 vs. control group.

To further clarify the mechanism by which CYP enhances antioxidant capacity in rats, the concentration of Nrf2 and the activation of MAPK ERK1/2 in the jejunum were detected with an ELISA kit and Western blot, respectively. The Nrf2 content in the jejunum was significantly elevated in the 0.5% CYP group compared with the control group (*P* < 0.05; Figure 1A). Moreover, CYP supplementation markedly increased the expression of phosphorylated ERK1/2 (p-ERK1/2) in the jejunum relative to controls (*P* < 0.05; Figure 1B). These findings suggest that CYP actives the MAPK ERK1/2 signaling pathway in the jejunum. Taken together, CYP may protect the jejunum from oxidative damage through the MAPK ERK1/2–Nrf2 signaling pathway; however, the precise underlying mechanisms warrant further investigation.

### 3.2 CYP promotes jejunum development in rats

**Figure 2:**
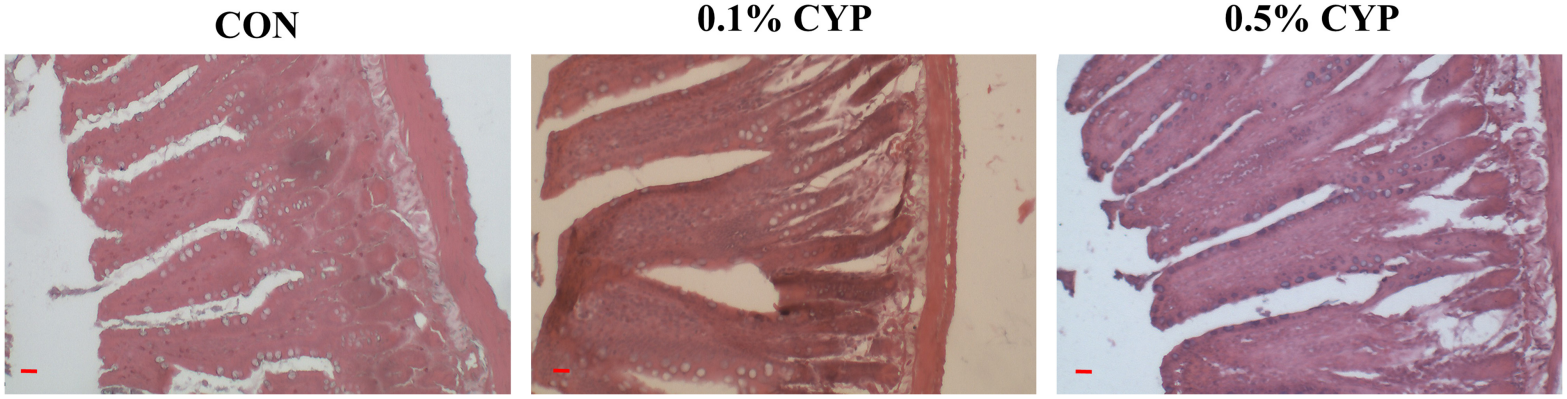
Histopathological analysis of jejunum in rats after dietary CYP supplementation. Representative H&E-stained jejunal sections showing villus height, crypt depth, and intraepithelial lymphocytes (IELs), scale bars are 10 μm. Compared with the control group, rats supplemented with 0.1 and 0.5% CYP exhibited taller and more intact villi, higher villus height-to-crypt depth ratios (V/C), and fewer IELs.

To evaluate the impact of CYP on jejunal development in rats, villus height, crypt depth, and the number of intraepithelial lymphocytes (IELs) were assessed in jejunal tissue. Compared with the control group, 0.1 and 0.5% CYP supplementation significantly increased villus height and the villus height-to-crypt depth ratio (V/C; *P* < 0.05), while reducing the number of intraepithelial lymphocytes (*P* < 0.05; Figure 2 and Table 5). These results indicated that CYP supplementation promotes jejunal morphological development and may contribute to improved intestinal health in rats.

**Table 5:** Effect of CYP on jejunal morphology of rats.

**Items**	**CON**	**0.1% CYP**	**0.5% CYP**	***P-*value**
Villus height (μm)	584.48 ± 23.13^b^	666.36 ± 19.30^a^	735.92 ± 27.91^a^	0.03
Crypt depth (μm)	171.50 ± 7.73	166.04 ± 42.46	176.52 ± 40.41	0.67
V/C	3.46 ± 0.39^b^	4.17 ± 0.22^a^	4.25 ± 0.28^a^	0.02
Epithelial lymphocyte number (%)	20.0 ± 3.68^a^	15.0 ± 1.2^b^	13.3 ± 2.7^b^	0.01

Within each row, values marked with different lowercase letters differ significantly (*P* < 0.05), while identical letters indicate no significant difference (*P* > 0.05).

### 3.3 CYP increases the activity of digestive enzymes in the jejunum of rats

To further investigate the effect of CYP on jejunal digestive capacity, the activities of key digestive enzymes were measured. Compared with the control and 0.1% CYP groups, the activities of lactase, sucrose, and maltase in the jejunum were significantly increased in the 0.5% CYP group (*P* < 0.05 or *P* < 0.01). In contrast, dietary CYP supplementation had no significant effect on Na^+^/K^+^-ATPase or Ca^2^^+^/Mg^2^^+^-ATPase activities (*P* > 0.05; Table 6). These results indicate that 0.5% CYP enhances carbohydrate digestive enzyme activity in the jejunum. Combined with morphological improvements, CYP supplementation appears to promote jejunal development and digestive function, thereby potentially improving nutrient digestion and absorption in rats.

**Table 6:** Effects of CYP on disaccharidase and ATPase activities in rat jejunum (U/mg protein).

**Items**	**CON**	**0.1% CYP**	**0.5% CYP**	***P*-value**
Lactase	7.08 ± 0.49^b^	8.31 ± 0.85^ab^	11.87 ± 0.92^a^	0.03
Sucrase	3.07 ± 0.37^Bb^	4.14 ± 1.05^ABab^	5.89 ± 0.74^Aa^	< 0.01
Maltase	427.40 ± 79.96^Bb^	550.60 ± 79.44^Bb^	875.60 ± 58.25^Aa^	< 0.01
Na^+^/K^+^-ATPase	0.62 ± 0.30	0.61 ± 0.40	0.63 ± 0.33	0.83
Ca^2+^/Mg^2+^-ATPase	0.53 ± 0.20	0.57 ± 0.24	0.72 ± 0.32	0.19

Within each row, values marked with different lowercase letters differ significantly (*P* < 0.05), those with different uppercase letters differ highly significantly (*P* < 0.01), while identical letters indicate no significant difference (*P* > 0.05).

### 3.4 CYP enhances jejunal immune function in rats

As presented in Table 7, supplementation with 0.5% CYP significantly increased the levels of IgA and DEFβ1 in the jejunum compared to the control and 0.1% CYP groups (*P* < 0.05 or *P* < 0.01). However, no significant changes were observed in IL-1β or IL-6 levels (*P* > 0.05). These results suggest that CYP enhances the local immune response in the jejunum, potentially contributing to improved intestinal barrier function in rats.

**Table 7:** Effects of CYP on immune response and mucosal barrier in the jejunum of rats (ng/mg protein).

**Items**	**CON**	**0.1% CYP**	**0.5% CYP**	***P*-value**
IgA	17.83 ± 1.42^b^	22.01 ± 0.64^b^	27.11 ± 1.92^a^	0.02
DEFβ1	58.91 ± 2.90^Bb^	69.14 ± 5.32^ABab^	75.40 ± 3.70^Aa^	< 0.01
IL-1β	22.91 ± 2.96	18.72 ± 1.58	21.04 ± 4.51	0.22
IL-6	19.87 ± 1.68	13.69 ± 2.69	19.16 ± 3.01	0.35

Within each row, values marked with different lowercase letters differ significantly (*P* < 0.05), those with different uppercase letters differ highly significantly (*P* < 0.01), while identical letters indicate no significant difference (*P* > 0.05).

### 3.5 CYP enhances jejunal intestinal barrier in rats

**Figure 3:**
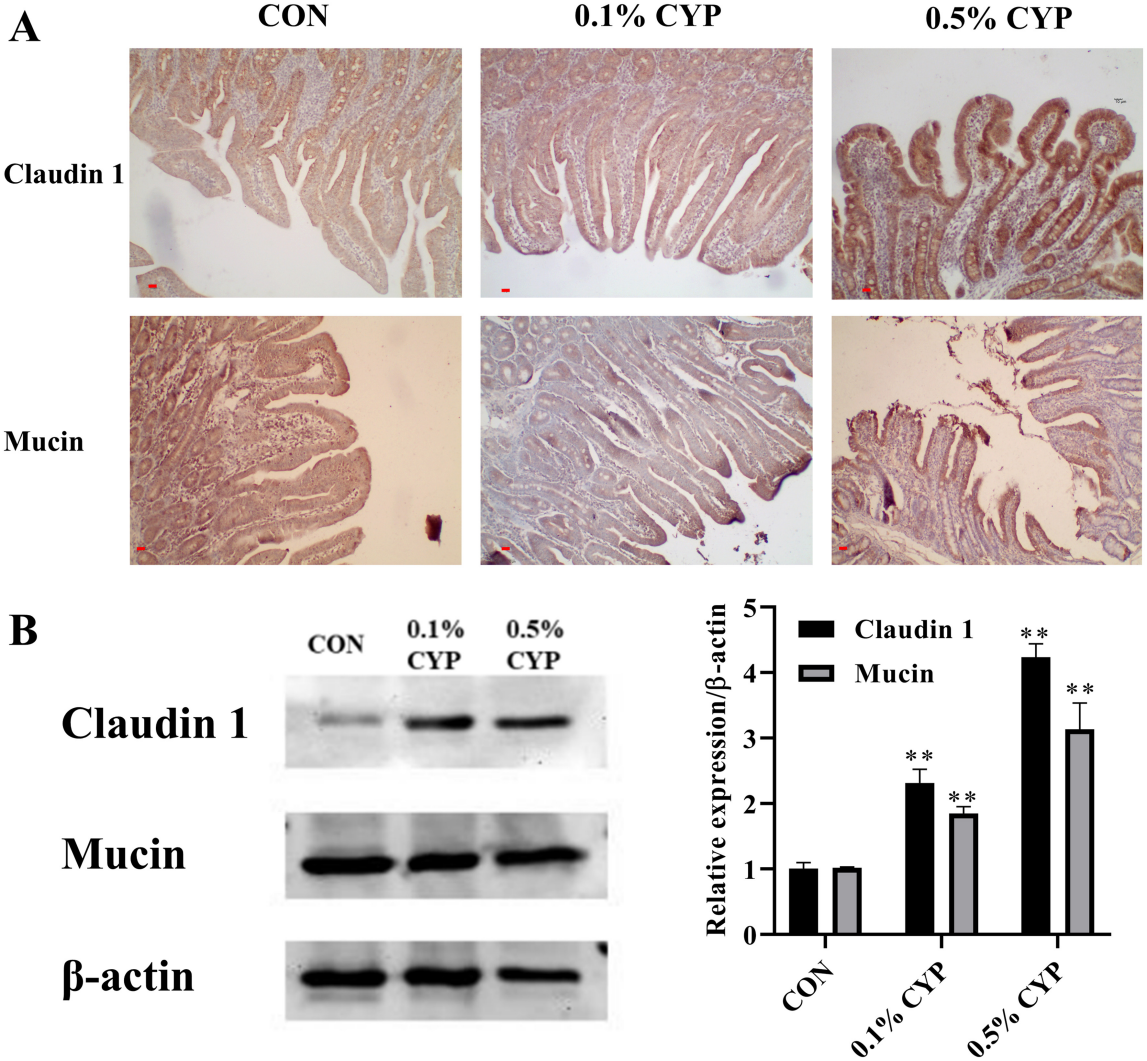
CYP promotes the intestinal barrier of jejunum in rats. The expression of Claudin 1 and mucin 1 in jejunum of rats was measured using IHC, scale bars are 10 μm **(A)** and Western blot **(B)**. ** denotes *P* < 0.01 vs. control group.

To evaluate the impact of CYP on the intestinal epithelial barrier, the expression of Claudin-1 and Mucin-1 in the jejunum was examined using immunohistochemistry (IHC) and Western blotting. As illustrated in Figure 3A, supplementation with 0.1 and 0.5% CYP markedly enhanced Claudin-1 and Mucin-1 expression in the jejunal epithelium compared with the control group. These results were corroborated by Western blot analysis, which showed significant increases in Claudin-1 and Mucin-1 protein levels in both CYP-treated groups (*P* < 0.05 or *P* < 0.01; Figure 3B). Collectively, these findings indicate that CYP promotes the integrity of the jejunal epithelial barrier, suggesting a potential mechanism by which it supports intestinal health in rats.

## 4 Discussion

The intestinal and immune systems of young animals are structurally and functionally immature, rendering them particularly vulnerable to environmental and dietary stressors that impair growth and health (21, 22). Early postnatal intestinal development depends on microbial colonization, which stimulates lymphocyte differentiation and the maturation of gut-associated lymphoid tissues, ultimately shaping a functional mucosal barrier (23). Given that the majority of immune cells reside in intestinal lymphoid tissues, maintaining intestinal integrity and immune homeostasis is essential for overall health (24, 25). In this context, plant-derived polysaccharides have attracted increasing attention as functional dietary components due to their immunomodulatory and antioxidant properties (26).

Oxidative stress is a key contributor to intestinal epithelial injury, as excessive reactive oxygen species (ROS) disrupt cellular membranes, organelles, and renewal processes (27). Natural polysaccharides are increasingly recognized as effective antioxidants capable of counteracting such damage (28). Previous studies have shown that Chinese yam polysaccharide (CYP) possesses strong free radical–scavenging activity and enhances antioxidant enzyme function in intestinal epithelial cells, potentially through activation of MAPK-related pathways (29, 30). MDA, a major lipid peroxidation product, reflects oxidative damage, while T-AOC represents overall antioxidant capacity (31). Nrf2 serves as a crucial transcription factor that preserves cellular redox homeostasis through the regulation of antioxidant gene expression (16, 32–35). In this study, CYP decreased serum MDA levels, increased T-AOC, elevated Nrf2 content, and activated the MAPK ERK1/2 signaling pathway in the jejunum of rats. These findings suggest that CYP may enhance jejunal antioxidant capacity via the MAPK ERK1/2–Nrf2 pathway, although the precise mechanisms require further investigation.

Beyond oxidative defense, intestinal morphology and digestive capacity are critical determinants of nutrient utilization and growth performance. Villus architecture reflects the absorptive surface area of the intestine, while digestive enzymes directly influence nutrient digestion and uptake. Although earlier studies have suggested that CYP can modulate gut microbiota and alleviate diarrhea (36, 37), its direct role in shaping intestinal structure and function has remained insufficiently characterized. The present findings support the notion that CYP contributes to improved intestinal functional capacity, likely by promoting epithelial development and enzymatic activity, thereby enhancing nutrient assimilation during the vulnerable weaning period.

Intestinal barrier integrity relies not only on epithelial structure but also on effective mucosal immune defenses. Secretory IgA and antimicrobial peptides such as defensins constitute key components of the first line of defense against luminal pathogens (38). Tight junction proteins and mucins further reinforce this barrier by regulating paracellular permeability and maintaining epithelial cohesion (39). The observed upregulation of Claudin-1 and Mucin-1 in response to CYP supplementation suggests that CYP may strengthen epithelial barrier function by coordinating immune protection with structural reinforcement, thereby reducing susceptibility to inflammation and pathogen invasion.

Collectively, this study provides evidence that dietary CYP exerts multifaceted protective effects on the jejunum of weaned rats by enhancing antioxidant defenses, supporting epithelial development, and reinforcing mucosal immune and barrier functions. These findings expand current understanding of the biological actions of CYP and highlight its potential as a functional feed additive for improving intestinal health during early life stages.

## Conclusion

In summary, dietary supplementation with 0.5% CYP significantly enhanced systemic antioxidant capacity, promoted jejunal structural development, increased digestive enzyme activities, and reinforced mucosal immunity and epithelial barrier integrity in weaned rats. These results indicate that CYP exerts multifaceted protective effects on intestinal function, likely through coordinated regulation of antioxidant defense and barrier-associated pathways. Collectively, this study supports the potential application of CYP as a natural functional feed additive to improve intestinal health and nutrient utilization during early life. Further investigations are warranted to elucidate the precise molecular mechanisms underlying these beneficial effects and to validate their applicability in other animal species.

## Data Availability

The raw data supporting the conclusions of this article will be made available by the authors, without undue reservation.
